# Clinical Effectiveness of Non-Immersive Virtual Reality Tasks for Post-Stroke Neuro-Rehabilitation of Distal Upper-Extremities: A Case Report

**DOI:** 10.3390/jcm12010092

**Published:** 2022-12-22

**Authors:** Debasish Nath, Neha Singh, Megha Saini, Onika Banduni, Nand Kumar, Madakasira Vasantha Padma Srivastava, Shanmugam Senthil Kumaran, Amit Mehndiratta

**Affiliations:** 1 Centre for Biomedical Engineering, Indian Institute of Technology Delhi (IITD), New Delhi 110016, India; 2 Department of Psychiatry, All India Institute of Medical Sciences (AIIMS), New Delhi 110029, India; 3 Department of Neurology, All India Institute of Medical Sciences (AIIMS), New Delhi 110029, India; 4 Department of Nuclear Medicine and Resonance, All India Institute of Medical Sciences (AIIMS), New Delhi 110029, India; 5 Department of Biomedical Engineering, All India Institute of Medical Sciences (AIIMS), New Delhi 110029, India

**Keywords:** case report, virtual reality, stroke, neuro-rehabilitation, distal upper extremities, cortical excitability, fMRI, DTI

## Abstract

A library of non-immersive Virtual Reality (VR) tasks were developed for post-stroke rehabilitation of distal upper extremities. The objective was to evaluate the rehabilitation impact of the developed VR-tasks on a patient with chronic stroke. The study involved a 50-year-old male patient with chronic (13 month) stroke. Twenty VR therapy sessions of 45 min each were given. Clinical scales, cortical-excitability measures, functional MRI (fMRI), and diffusion tensor imaging (DTI) data were acquired pre-and post-therapy to evaluate the motor recovery. Increase in Fugl-Meyer Assessment (wrist/hand) by 2 units, Barthel Index by 5 units, Brunnstrom Stage by 1 unit, Addenbrooke’s Cognitive Examination by 3 units, Wrist Active Range of Motion by 5° and decrease in Modified Ashworth Scale by 1 unit were observed. Ipsilesional Motor Evoked Potential (MEP) amplitude (obtained using Transcranial Magnetic Stimulation) was increased by 60.9µV with a decrease in Resting Motor Threshold (RMT) by 7%, and contralesional MEP amplitude was increased by 56.2µV with a decrease in RMT by 7%. The fMRI-derived Laterality Index of Sensorimotor Cortex increased in precentral-gyrus (from 0.28 to 0.33) and in postcentral-gyrus (from 0.07 to 0.3). The DTI-derived FA-asymmetry decreased in precentral-gyrus (from 0.029 to 0.024) and in postcentral-gyrus (from 0.027 to 0.017). Relative reduction in task-specific performance metrics, i.e., time taken to complete the task (31.6%), smoothness of trajectory (76.7%), and relative percentage error (80.7%), were observed from day 1 to day 20 of the VR therapy. VR therapy resulted in improvement in clinical outcomes in a patient with chronic stroke. The research also gives insights to further improve the overall system of rehabilitation.

## 1. Introduction

Residual upper-arm disabilities are common morbidities in the chronic phase of recovery, affecting more than 66% of patients with stroke [1]. The literature suggests the requirement of intensive practice to facilitate functional recovery in the chronic phase [2]. However, factors such as lack of objective assessment, associated clinical burden and boredom highly limit the effectiveness of traditional rehabilitation [3]. In recent years, Virtual Reality (VR)-assisted rehabilitation has emerged as a supplementary approach to address some of the limitations associated with traditional physiotherapy [4,5]. Post-stroke rehabilitation of distal upper extremities is comparatively slower and requires intensive targeted practice to conduct Activities of Daily Living (ADL) [6]. However, limited literature exists to support targeted VR-based rehabilitation of distal upper extremities [7]. Furthermore, the exact neurophysiological aspects showing enhanced post-stroke recovery at the neuronal level are still unclear [8]. In our previous study, a library of VR tasks specific to distal-joint rehabilitation was developed, and task-specific outcome metrics were validated with forty healthy subjects and two patients with stroke [9]. In this study, we present the case of a 50-year-old male patient with 13-month chronic stroke who participated in VR therapy and the associated functional and neuronal changes observed in response to the therapy. The primary objectives of this study were to evaluate the efficacy of the developed VR tasks and to investigate the neurophysiological behavior supporting post-stroke motor recovery in response to the VR therapy during the chronic phase. 

## 2. Case Description

The study was approved by the Institutional Review Board (IRB) at All India Institute of Medical Science (AIIMS), New Delhi, India, under protocol-number IEC-229/11.4.2020. The patient provided written informed consent before enrollment in this study. 

### 2.1. Subject

**Figure 1:**
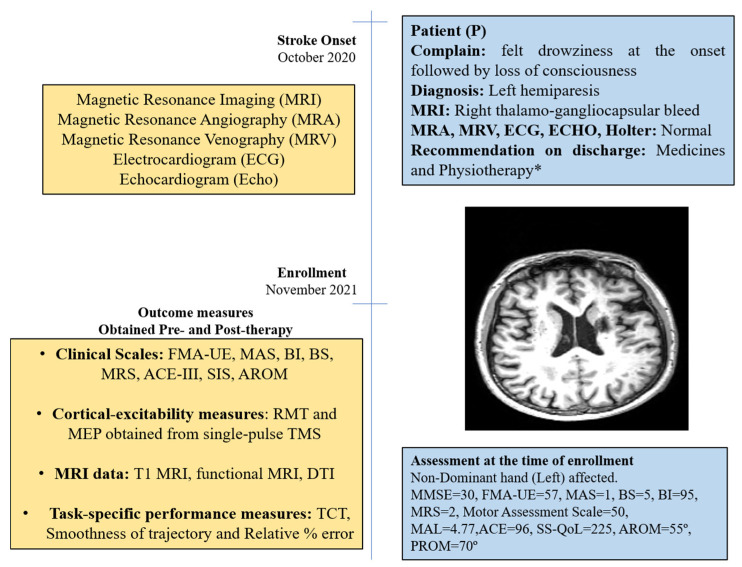
Timeline with relevant data from the episodes of care and clinical assessment scores obtained at the time of enrollment. * Details in Supplementary Material.

The patient was a 50-year-old right-handed male (henceforth, referred as “P”); he was well educated and was a lawyer by profession. He had an incidence of right thalamo-gangliocapsular-bleed in October 2020 (Supplement Figure S1). Additional clinical-presentation details are provided in the Supplementary Materials (Section S1). He had no history of substance abuse, such as consuming tobacco or alcohol, but experienced hypertension in the past 10 years and diabetes for past 1 year. After onset of stroke, he was admitted to a local hospital and discharged after 14 days for rehabilitation management at home. He underwent supervised home-based physiotherapy after 3 months after stroke-onset (see Supplementary Materials). Assessment scores at enrollment are given in Figure 1.

### 2.2. Therapy Protocol

#### 2.2.1. VR Task Development

**Figure 2:**
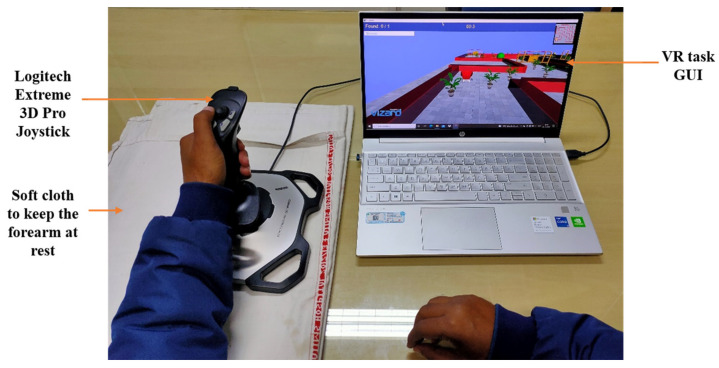
VR therapy setup.

In our previous work, a library of joystick-based non-immersive VR tasks specific to rehabilitation of the distal upper extremity were developed and validated [9]. The hand motions were mapped into the VR-environment displayed on a computer screen using a joystick (Logitech Extreme 3D Pro, Lausanne, Switzerland) interface (Figure 2). With ±15° of yaw and pitch angle from its mean position, the joystick was used to train wrist flexion/extension and wrist radial/ulnar deviation motion. The front button of the joystick was used for index finger flexion/extension, while buttons on the top of the joystick were used for thumb abduction/adduction motion. The designed VR task requires the user to reach a pre-defined target position, travelling through a track with obstacles given at a reasonable time interval (Figure 2). The VR framework consists of two different environments: (i) Individual Environment (IE) and (ii) Combined Environment (CE). IE tasks have a single factor of difficulty. CE tasks were designed in a similar environment as IE but have multiple factors of difficulties simultaneously to increase the complexity of the task and rehabilitation training for the user (details in Supplementary Table S1). Different motor and cognitive tasks with graded difficulty levels were designed over multiple shapes of tracks [9] (details in Supplement Section S2.2). A collision with the obstacles results in an error with audio feedback. Appropriate audio-visual feedback appears as per the performance of the user after completing a task.

#### 2.2.2. Healthy Subjects

Three performance metrics—time taken to complete the task (TCT), smoothness of trajectory, and relative percentage error—were defined and validated with forty healthy subjects in our previous work [9], which is treated as a reference in this study for comparison. A detailed explanation and definition of the metrics are given in the Supplementary Materials (Data acquisition Section S3.3).

#### 2.2.3. Therapy Sessions

VR therapy was given for 20 sessions of 45 min of therapy (with 15 min of rest period) in the presence of an experienced physiotherapist. The patient was asked to comfortably sit straight on a chair and place their arm on the table, with their elbow at ~120 degrees (Figure 2). The patient was instructed to control the joystick movements using their wrist and fingers, with limited involvement of any proximal joints. A pillow/soft cloth was used for the purpose of keeping the forearm fixed, to minimize any compensatory movements.

### 2.3. Patient Data Acquisition

#### 2.3.1. Subjective and Objective Scales

Clinical scales, including the Modified Ashworth Scale (MAS), Modified Rankin Scale (MRS), Fugl-Meyer Assessment (FMA), Stroke Impact Scale (SIS), Motor Assessment Scale, Barthel Index (BI), Brunnstrom Stage (BS), Motor Activity Log (MAL), Addenbrooke’s Cognitive Examination (ACE-III), and Active and Passive ROM were used at day 1 (before the start of session) and day 21 (a day after the last session). MAS was used to measure the degree of muscle spasticity, BS and MRS to identify stages of stroke recovery, Motor Assessment Scale and FMA to evaluate the motor control and functioning, BI and MAL to assess ADL performance, and SIS and Stroke-Specific Quality of Life (SS-QoL) to evaluate the health-related quality of life as per the patient’s perspective. In addition, a self-designed subjective feedback form (SQF), System Usability Scale (SUS), and Visual Analogue Scale-Fatigue (VAS-F) were administered at day 21 to obtain the patient’s experience of the VR therapy.

#### 2.3.2. Cortical Excitability Measures

P was compatible with Transcranial Magnetic Stimulation (TMS). Single-pulse TMS stimuli were given at 100% Motor Threshold in accordance with the procedure widely used [10] (details in Supplementary Section S3.1) using a figure-of-eight coil (type D70 (AC), Magstim Rapid^2^, Whitland, UK) from the Extensor Digitorum Communis (EDC) muscle.

#### 2.3.3. Magnetic Resonance Imaging (MRI)

Anatomical T1 image, Diffusion Tensor Image (DTI), and Blood Oxygenation Level Dependent (BOLD) functional MRI (fMRI) images (for both the affected and unaffected hand movements) were acquired using a 3T MR Scanner (Philips Ingenia 5.7.1). Data acquisition and analysis steps are given in Supplementary Materials Section S3.2.

## 3. Clinical Rehabilitation Impact

The therapy protocol proceeded as planned, and the patient tolerated the therapy without any adverse events.

### 3.1. Clinical Scores

Clinical scores obtained pre- and post-therapy are given in Table 1. The functional gains were observed with the increase of the FMA-UE score by 4 units. A decrease in spasticity was observed from the reduction in MAS values from 1 to 0 at both the wrist and fingers. BI, BS, and Motor Assessment Scale scores increased post-therapy, with no change in MRS and MAL scores (Table 1). Post-therapy active range of motion (AROM) at the wrist increased by 5°. The SS-QoL score increased by 5 units pre- to post-therapy. Increased values in SIS domains of strength, emotion, ADL/IADL, mobility, and hand function were observed (see Supplementary Sections S1.3 and S1.5). ACE-III attention and memory domain scores increased by 1 and 2 units, respectively (Supplement Sections S1.3 and S1.5).

### 3.2. Cortical Excitability Measures

Pre- to post-therapy, in more than 5 out of 10 consecutive trials, the ipsilesional Resting Motor Threshold (RMT) reduced by 7%, and the Motor Evoked Potential (MEP) amplitude increased by 60.9 µV, with observed muscle contraction response in the dorsal wrist and third digit. Similarly, a 7% decrease in the contralesional RMT and 56.2 µV increase in MEP amplitude were obtained, with muscle contraction response in the dorsal wrist (Table 1). Considering the relative change (ratio of difference between post- and pre- therapy scores normalized to pre-therapy scores in %), MEP amplitudes showed an increase of 68.4% for the ipsilateral hemisphere and an increase of 63.1% for the contralateral hemisphere. Considering the relative percentage change in RMT, a decrease of 10.7% and 11.7% were observed for the ipsilateral and contralateral hemisphere, respectively.

### 3.3. fMRI measures

**Figure 3:**
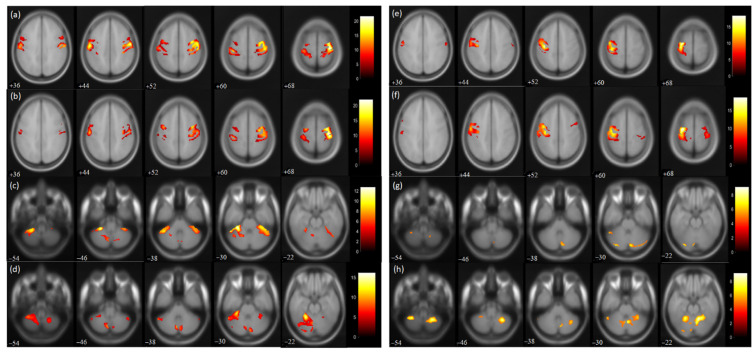
BOLD functional MR images for wrist extension task with voxel level threshold *p* < 0.05 (FWE corrected) and cluster level threshold of 5 voxels. Talairach-client was used to correlate MNI coordinates with gray and white matter. (**a**,**c**) pre-therapy activations and (**b**,**d**) post-therapy activations during affected hand trial; (**e**,**g**) pre-therapy activations and (**f**,**h**) post-therapy activations during unaffected hand trial for the masked regions of sensorimotor cortex (36:8:68 slices) and cerebellum (−54:8:−22 slices).

For the affected hand trial, the number of activated voxels was observed to be reduced in the post-therapy compared to the pre-therapy acquisition (Figure 3). The activated voxels in the contralateral (ipsilesional) sensorimotor cortex (SMC), i.e., pre-central (from 1695 to 1248) and post-central gyrus (from 1549 to 1159), were observed to be decreased (Table 2). Similarly, the number of activated voxels in the contralateral supplementary motor area (SMA) was also observed to be decreased post-therapy (from 703 to 301) (Table 2). A similar reduction was observed in the ipsilateral (contralesional) SMC pre-central (from 946 to 619) and post-central gyrus (from 1349 to 619). Similarly, ipsilateral SMA activation was also observed to be decreased (from 619 to 245). The value of the Laterality Index (LI: given as ratio of [contralateral voxels − ipsilateral voxels]/[ contralateral voxels + ipsilateral voxels]), ranging from −1 (all ipsilateral/contralesional activation) to 1 (all contralateral/ipsilesional activation), was found to be increased in SMC pre-central (from 0.28 to 0.33) and post-central gyrus (from 0.07 to 0.3) and in SMA (from 0.06 to 0.10). For trial with the affected hand, the number of activated voxels was increased in the ipsilateral cerebellum (CBM) exterior (from 1296 to 2049) and decreased in the contralateral CBM exterior (from 980 to 203). For the affected hand trial, an increase in ipsilateral cerebellum ratio (given as ratio of [ipsilateral cerebellum voxels]/[ ipsilateral cerebellum voxels + contralateral cerebellum voxels) was observed for the CBM exterior (from 0.56 to 0.91).

For the trial with the unaffected hand, the number of activated voxels was increased in the contralateral/contralesional SMC, i.e., pre-central (from 1362 to 1428) and post-central gyrus (from 808 to 1106). Similarly, contralateral SMA activation was found to be increased (from 71 to 375). Increased activation was also observed in the ipsilateral/ipsilesional SMC, i.e., pre-central (from 18 to 77) and post-central (from 73 to 208) gyrus. Ipsilateral SMA activation was also increased (from 61 to 260). The ipsilateral cerebellum ratio decreased in the CBM exterior (from 0.79 to 0.71). The LI values of SMC were found to decrease in the pre-central (from 0.97 to 0.89) and post-central gyrus (from 0.83 to 0.68) post-therapy. Similarly, the LI of SMA was found to be reduced (from −0.07 to −0.18) post-therapy.

### 3.4. DTI Measures

**Figure 4:**
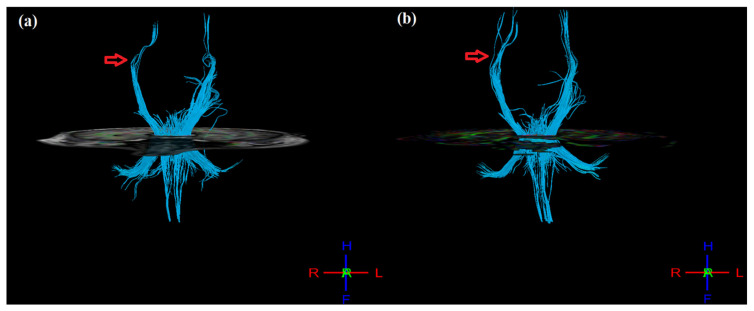
3D tractography images of CST of both hemispheres (**a**) pre- and (**b**) post-therapy. Post-therapy image of affected (right) hemisphere indicating denser and more intact tracts as compared to pre-therapy (indicated by red arrow mark).

Post-therapy, mean FA values were calculated for masked regions of pre-central gyrus, post-central gyrus, thalamus, and cortico-spinal tract (CST) (Table 3). In the ipsilesional hemisphere, FA values increased for the pre-central gyrus (from 0.346 to 0.368), post-central gyrus (from 0.342 to 0.367), thalamus (from 0.425 to 0.454), and CST (0.608 to 0.615). Similarly, in the contralesional hemisphere, FA values increased in the pre-central gyrus (from 0.368 to 0.386) and post-central gyrus (from 0.367 to 0.380) but decreased in the thalamus (from 0.454 to 0.393) and CST (from 0.614 to 0.600). However, FA asymmetry (aFA: given as [FA_contralesional_ − FA_ipsilesional_]/[FA_contralesional_ + FA_ipsilesional_]), ranging from −1 to 1, decreased post-therapy for the pre-central gyrus (from 0.029 to 0.024), post-central gyrus (from 0.027 to 0.017), thalamus (from −0.037 to −0.072), and CST (from 0.005 to −0.012). In addition, the 3D tractography result shows improvement in CST, where the post-therapy CST of the ipsilesional (right) hemisphere appear denser and more intact as compared to pre-therapy (Figure 4), as supported by the corresponding changes observed in aFA values.

### 3.5. Task-Specific Performance Measures

**Figure 5:**
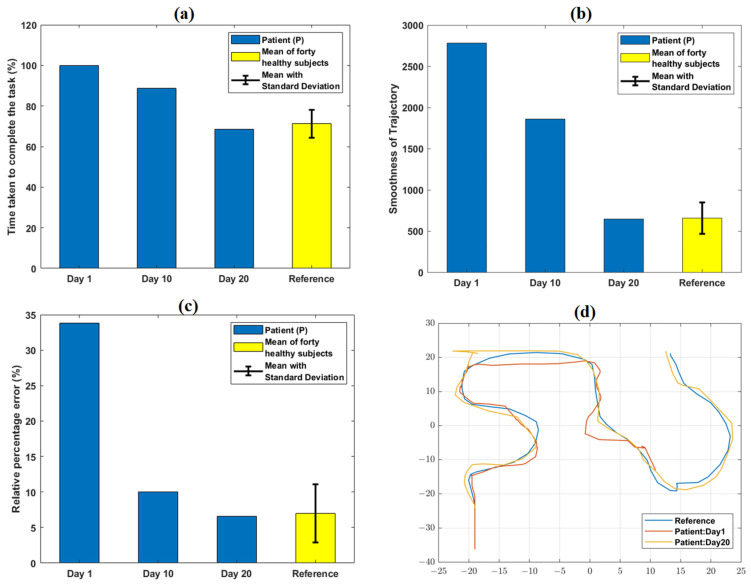
Variation in quantitative task-specific performance metrics: (**a**) TCT, (**b**) smoothness of trajectory, and (**c**) relative % error obtained from the patient (P) at day 1, 10, and 20 of VR therapy. A relative decrease in TCT, smoothness of trajectory, and relative % error values was observed from day 1 to day 20. The reference values shown were obtained from the average performance metrics of 40 healthy subjects in our earlier work. (**d**) shows qualitative trajectory plots for day 1 and 20 obtained from P. At day 1, the trajectory of P was incomplete; it became identical with that of a representative healthy subject (obtained from our previous work) at day 20.

The comparison of task-specific performance metrics for a representative task level (CE4-L1) obtained on day 1, 10, and 20 are shown in Figure 5. TCT values relatively decreased from day 1 (100%) to day 20 (68.4%) of the VR therapy (Supplementary Table S2). Similarly, smoothness of trajectory and relative % error values at day 20 decreased by 76.7%, and 80.7%, respectively, compared to day 1 (Supplementary Table S2). Figure 5d and Supplementary Figure S2 graphically indicate the trajectory path of P obtained on day 1 and 20 of VR therapy.

### 3.6. Subjective Questionnaire Feedback (SQF)

In order to obtain the patient’s experience with VR therapy, a subjective questionnaire feedback form (SQF) was administered at day 21 (Supplementary Table S3). P found the VR tasks enjoyable, easy to understand, and motivating to perform. He found VR safe and easy to use and showed interest in using the setup at home, provided with the detailed instructions. He had no difficulty in understanding the protocol and performing accordingly. With no earlier VR exposure, he took one to two task sessions to be comfortable with the therapy protocol (Q22, SQF; Supplementary Table S3). He experienced a very low degree of visual fatigue, as evaluated by VAS-F score (46 out of 180). He became fatigued easily during the initial sessions, but gradually grew accustomed to tasks as the sessions progressed. In response to the questions regarding “*how to improve the system for further use?*” (Q30, SQF; Supplementary Table S3), he suggested incorporation of different 3D models and graphics in the VR environment to make it more interesting and enjoyable. He preferred to use a soft cloth to rest his forearm. In addition, he suggested altering the placement of buttons on top of the joystick for more convenient usage. The post-therapy product usability as evaluated by SUS had a score of 80.

## 4. Discussion

The developed VR framework demonstrated improved outcomes in terms of clinical and neurophysiological changes in a chronic stroke-survivor. In addition to improvements observed in the clinical scales, such as FMA-UE, MAS etc., both ipsilesional and contralesional RMT decreased (~10%) and MEP increased (~60%). Ipsilateral BOLD-fMRI activation in the pre-and post-central gyrus reduced post-therapy, with increased activation in the ipsilateral cerebellum. DTI measures such as ipsilesional FA values increased in the pre- and post-central gyrus and thalamus, with a decrease in aFA ratio.

### 4.1. Changes in Clinical Scores and Cortical-Excitability Measures

P demonstrated a reduction in upper limb impairment as evidenced by the improvements in clinical scales. An increase in the wrist/hand components of FMA-UE (2 units) indicated an enhancement of distal upper-arm functionality post-therapy. A post-therapy increment has been observed in proximal (S/E) component of FMA-UE, possibly because of their involvement as a compensatory mechanism during distal training [7,11]. Reduction of spasticity at wrist and fingers has been reflected with smoother and better wrist and finger activities with therapy progression, resulting in better completion of the tasks. Post-therapy, the patient was able to use his thumb and index finger without any fatigue and was able to pick and place small objects in an improved manner. The increment in AROM at the wrist (5°) indicated an increment in degree of movement of the wrist joint as involved in ADLs with ease, as supported by increased BI-score. Increase in SIS domains: hand function, mobility, emotions, and strength suggested a relative increase in Health-Related Quality of Life (HRQoL) from the patient’s perspective. The requirement of constant attention and ability to remember the button sequence for successful completion of the cognitive task levels might be the possible reason for the increase in attention and memory domains of ACE-III.

The changes observed in the cortical excitability in both the hemispheres in terms of reduction in RMT and increment in MEP amplitude for the EDC muscle cortical representations suggest improvement in neuroplasticity and motor-cortex excitability [12,13]. RMT refers to the threshold of the pyramidal tract’s response to magnetic stimulus, indicating the neuronal membrane excitability [14]. Neuronal excitability decreases after stroke, exhibiting higher RMT [15], which should decrease for the ipsilesional hemisphere post-therapy, indicating enhanced cortical excitability [16,17,18,19]. As the functional recovery for post-stroke patients primarily depends on the integrity of the corticospinal tract, the observed cortical excitability suggests a potential restoration and improvement of these tracks [20,21].

### 4.2. Changes in fMRI Activations and DTI-derived Measures

A reduction in BOLD-fMRI intensity was observed in both hemispheres. In a typical healthy subject, for hand movements, the hemisphere contralateral to that hand is more activated [22]. However, post-stroke patients exhibit a greater ipsilateral/contralesional activation with affected hand movements [23,24]. Pre-therapy LI of SMC observed in the pre-central and post-central gyrus was close to zero, indicating bilateral cortical activation (Figure 3). However, post-therapy LI values of SMC increased (0.17 to 0.29, Δ = 0.12) and were close to 1, indicating lateralization towards the contralateral hemisphere (Table 2). The results agree with previous studies indicating motor recovery [25,26,27]. Similarly, the LI value of SMA increased (Δ = 0.04) from pre-to post-therapy, which might be attributed to the role of SMA in planning and rehearsal of movement sequences and is consistent with previous studies [27,28]. Contralesional activation has been found to be reduced post-therapy, most probably due to the brain relearning process, as reported earlier [29,30,31]. The literature has explained the evolved contralesional activation after stroke as a compensatory approach in response to task difficulty [32], which might decrease with skill acquisition and voluntary control with repeated practice [33]. Significant increase in the activation of the ipsilateral CBM exterior during affected hand trials might indicate motor recovery attributed to the role of CBM in motor learning [34,35,36]. Increase in the ipsilateral cerebellum ratio (Δ = 0.35) for the affected hand trial could be another indication of improved cerebellar–cerebral functional connectivity [34,37].

Earlier studies have reported that due to axonal degeneration, FA values decline in the lesioned hemisphere for post-stroke patients [38,39], resulting in increased interhemispheric aFA values [40]. Lower interhemispheric aFA has been found to be correlated with higher upper-limb FMA score and hence with better motor recovery [41,42], and vice versa [43,44]. Therefore, in our case, the post-therapy results indicating increased FA values in the lesioned hemisphere with decreasing interhemispheric aFA values reflect a better integrity of the affected tracts, the same as reported in the literature [45].

### 4.3. Changes in Task-Specific Performance Measures

At day 1, P failed to complete the particular task level within the given time boundary. Hence, the TCT reached its maximum value of 100% (as per the equation defined in Supplement Section S3.3) at day 1 (Figure 5). With therapy progression, because of better task learning and repeated familiarization with the VR environment, P took 31.4% less time at day 20 with respect to day 1 to successfully reach the target. Therefore, TCT at day 20 decreased to 68.4% with respect to its maximum value. The variation of the patient’s performance in terms of TCT with the reference (mean ± SD = 71.2% ± 6.8%) obtained from forty healthy subjects (from [9]) is shown in Figure 2a.

At day 1, the value of trajectory smoothness was considerably higher (2783.2) than that of the reference value (659) obtained from forty healthy subjects [9]. Repeated practice and task familiarization resulted in reduced TCT and successful achievement of the targeted distance. Therefore, the smoothness parameter further decreased and attained value (648) numerically closer to the reference [9] with therapy progression. At day 1, P was unable to reach the target within the provided timeframe, resulting in a higher (33.8%) relative % error as compared to the reference (6.9%) [9]. However, being able to optimally reach the target as the sessions progressed, the parameter (6.5%) attained value closer to the range of reference.

The qualitative trajectory plots (Figure 5d) were used to subjectively evaluate the patients’ performances at day 1 and 20. Comparing these plots with that of a healthy subject can give information about the motion quality, such as incompleteness or any wrong path followed. Clinical significance of the improvements in terms of the performance metrics can be inferred, i.e., lower TCT and smoothness parameters might be an indication of better focus and coordination. Similarly, a lower relative % error might reflect improved precision in reaching the target. The defined task-specific performance metrics indicating pre-to post-therapy changes might be useful for further customizing the protocol as per a patient’s exact requirements, modulating therapy difficulty level, and monitoring a patient’s progress quantitatively.

The SUS score of 80 for this case, along with the obtained subjective feedback (Supplementary Table S1), demonstrated potential good acceptability of the VR setup in a clinical setting. Comparing with the available literature on targeted distal upper-limb VR rehabilitation, the study by Shin et al. [7] demonstrated a pre- to post-therapy change in the FMA-UE W/H component (~1.8) and SIS (~20) for the experimental group. Similarly, a VR-assisted cognitive rehabilitation study by Faria et al. [46] showed an increase in total ACE-III (~9) score post-intervention. Limited studies have explored the cortical excitability parameter changes from pre- to post-VR upper-limb rehabilitation [12,13]. A pilot study using glasses-free upper-limb VR-rehabilitation showed a 28.98% increase in pre- to post-MEP amplitude of the experimental group after a 3-week session [47]. Earlier studies have validated task-specific performance measures such as TCT [5] and smoothness of trajectory [48] for a group of healthy subjects without any patient-specific data.

The main objective of this case study was to evaluate the clinical effectiveness of the developed VR tasks specific to upper-limb distal joints. The developed VR protocol has shown potential in terms of the improvement in clinical scales, cortical excitability measures, task-specific performance metrics, and promotion of neuroplasticity through fMRI and DTI results. As reported, the post-stroke recovery during the chronic phase is less spontaneous; it depends mostly on the level of activity [49]. The developed VR setup is in its initial phase, further changes are to be incorporated to make it more specific, acceptable, and customizable for a large cohort of patients with stroke. This case study provided initial evidence of its potential for post-stroke distal upper-limb rehabilitation. Therefore, it is important to further evaluate the effect and contribution of VR therapy in addition to the current care of practice, conventional physiotherapy. The strength of this case study lies in the exploration of multi-dimensional aspects of stroke recovery, including changes in clinical measures and different neurophysiological aspects (cortical excitability measures, neuroimaging data such as fMRI and DTI) in response to a distal upper-limb-specific VR-assisted rehabilitation training for a chronic stroke-survivor.

### 4.4. Limitations and Future Scope and Future Scopes

This study has some limitations. The study lacks mid-term assessment of the clinical scales. In addition, a long-term follow-up evaluation was not performed. As the study uses a non-immersive VR setting, the degree of immersion was not evaluated. The used joystick has some inherent limitations, such as limited ROM and lack of a button customization option for ease of use. There are several ways to further improve the study with respect to the limitations mentioned. The addition of a head mounted display (HMD) device could provide the patient with an enriched environment to practice and hence possibly a better engagement in the therapy. Addition of activity-based clinical scales such as Action Research Arm Test (ARAT), Wolf Motor Function Test (WMFT), nine-hole pegboard test (9-HPT) etc. could give insights into the functional recovery in response to the therapy. We are in process to develop an in-house built joystick having a higher ROM, appropriate button placements for comfortable use and incorporation of biofeedback such as Electromyogram (EMG) in the VR-setup to make it customizable with a more patient-centric approach. In the future clinical trial, more patients including acute, sub-acute and chronic stages are to be recruited with a control group to evaluate any differences in rehabilitation outcomes, with mid-term clinical assessments and long-term follow-ups.

### 4.5. Implications of Using VR in Clinical Practice

Novel technologies such as VR may provide high level of patient engagement through an enriched experience. It also provides opportunities to practice individual patient at their own comfort and controls several other relevant factors such as number of repetitions, intensity of difficulty level and task-oriented targeted training, facilitating neuroplastic changes during rehabilitation. In addition, unique features of VR technology such as quantitative assessment and easy-to-setup for home-based training will enable remote monitoring of patients’ prognosis thereby reducing clinical burden at a resource-limited settings. VR technology thus holds very promising translational potential to clinical practice for rehabilitative use.

## 5. Conclusions

The developed VR-task has shown its potential to promote the recovery of distal upper-extremities during the chronic-phase of stroke in terms of various outcome measures such as: clinical scales, cortical excitability measures, neuroimaging derived outcomes, and task-specific performance outcomes. The observed clinical improvements demonstrated potential of practice-induced neuroplasticity in a chronic stroke-survivor in response to the non-immersive VR-based therapy. The preliminary results observed from this case study were encouraging and therefore, needs to be validated with a larger patient-cohort for further customization and implementation in clinical-settings.

## Figures and Tables

**Table 1:** Details of clinical scales and cortical excitability measures acquired from patient ‘P’.

Clinical Scales	Patient (P)	
	Pre-Therapy	Post-Therapy
FMA-UE	57	61
FMA-UE(W/H)	22	24
FMA-UE(S/E)	35	37
MAS (both wrist and fingers)	1	0
BS	5	6
MRS	2	2
BI	95	100
Motor Assessment Scale	50	53
AROM	0°–55°	0°–60°
PROM	0°–70°	0°–70°
MMSE	30	-
VAS-F	-	46
SUS	-	80
MAL	4.77	4.77
SS-QoL	225	230
Ipsilateral MEP (µV)	89.06	149.99
Ipsilateral RMT (%)	65	58
Contralateral MEP (µV)	89.06	145.31
Contralateral RMT (%)	60	53

Note: FMA-UE (max 66): Fugl-Meyer Assessment-Upper Extremity; FMW/H (max 24): Wrist/Hand component of FMA; S/E (max 42): Shoulder/Elbow component of FMA; MAS (0–4): Modified Ashworth Score; BS (max 7): Brunnstrom Stages; BI (max 100): Barthel Index; MRS (max 5): Modified Rankin Scale; Passive ROM (max 70°); Active ROM (max 70°); MMSE (max 30): Mini-Mental State Examination; SIS (max 100%): Stroke Impact Scale; SUS (max 100): System Usability Scale; VAS-F (max 180): Visual Analogue Scale-Fatigue; SS-QoL (max 230): Stroke Specific Quality of Life; MAL (max 5): Motor Activity Log; MEP: Motor Evoked Potential (in µV); RMT: Resting Motor Threshold (in %, max 100).

**Table 2:** Pre- and Post-VR Blood Oxygen Level Dependent (BOLD) activation pattern in affected and unaffected hand trials in masked regions of patient ‘P’.

P: Task by Affected Hand										
Region	PRE					POST				
	No. of Voxels	Threshold	No. of Voxels	Threshold	LI/IpsCB Ratio	No. of Voxels	Threshold	No. of Voxels	Threshold	LI/IpsCB Ratio
Hemisphere	Right		Left			Right		Left		
PrG	1695	21.66	946	17.43	0.28	1248	22.01	619	13.75	0.33
PoG	1549	19.68	1349	19.07	0.07	1159	21.23	619	13.75	0.303
CBM Exterior	980	9.66	1296	12.73	0.56	203	9.84	2094	16.17	0.911
SMA	703	14.48	619	15.2	0.06	301	14.79	245	12.36	0.102
**P: Task by Unaffected Hand**										
PrG	18	5.59	1362	18.35	0.97	77	7.7	1428	18.56	0.89
PoG	73	8.02	808	15.21	0.834	208	8.12	1106	14.22	0.68
CBM Exterior	271	8.24	72	9.11	0.79	443	9.16	180.	8.32	0.71
SMA	61	7.16	71	9.28	−0.075	260	8.32	375	11.52	−0.181

Note: PrG: Pre-central gyrus; PoG: Post-central gyrus; SMA: Supplementary Motor Area; CBM: Cerebellum; Laterality Index (LI) = (contralateral voxels − ipsilateral voxels)/(contralateral voxels + ipsilateral voxels); Ipsilateral cerebellum (Ips CBM) ratio = ipsilateral cerebellum voxels/(ipsilateral cerebellum voxels + contralateral cerebellum voxels).

**Table 3:** Mean FA and aFA values obtained from pre-and post-VR therapy DTI in masked regions of patient ‘P’.

Region	Right (Ipsilesional Hemisphere)		aFA	Left (Contralesional Hemisphere)		aFA
	Mean FA Values			Mean FA Values		
	PRE	POST		PRE	POST	
PrG	0.346	0.368	0.029	0.367	0.386	0.024
PoG	0.342	0.367	0.027	0.361	0.380	0.017
Thalamus	0.425	0.454	−0.037	0.395	0.393	−0.072
CST	0.608	0.615	0.005	0.614	0.600	−0.012

Note: FA: fractional anisotropy; FA asymmetry (aFA) = (FA_contralesional_ − FA_ipsilesional_)/(FA_contralesional_ + FA_ipsilesional_); PrG: pre-central gyrus; PoG: post-central gyrus; CST: cortico-spinal tract.

## Data Availability

The datasets used in this study are available from the corresponding author on reasonable request.

## Supplementary Materials

The following supporting information can be downloaded at: https://www.mdpi.com/article/10.3390/jcm12010092/s1.
