# Supplementary material for: Clinical Effectiveness of Non-Immersive Virtual Reality Tasks for Post-Stroke Neuro-Rehabilitation of Distal Upper-Extremities: A Case Report
Source: J Clin Med. 2022 Dec 22;12(1):92. doi: 10.3390/jcm12010092 (PMC9820917; doi:10.3390/jcm12010092)
Supplement: Supplementary file 1 [file jcm-12-00092-s001.zip › jcm-2059684-supplementary.pdf]

Journal of
Clinical Medicine

MDPI

Supplementary Material

Clinical Effectiveness of Non-Immersive Virtual Reality Tasks for Post-Stroke Neuro-Rehabilitation of Distal Upper-Extremities: A Case Report

S1. Patient (P) Details:

Age: 50 (at the time of enrollment)

Sex: Male

Handedness: Right

Occupation: Lawyer

Family history of stroke: His Elder brother

Relevant genetic or psychosocial history: None

S1.1. Details at the Onset of Stroke (October 2020):

Complain: Drowziness at stroke onset

Diagnosis: left hemiparesis

BP: 120/80, *Pulse rate:* 80/min, *Left Limb power:* 3-/5

MRI: Right thalamo-gangliocapsular bleed

MRA, MRV, ECG, ECHO, Holter: Normal

State: Drowzy

History of hypertension, diabetes, tobacco smoking or alcohol: hypertension (from past 10 years) and diabetes

Prior Transient Ischemic attack (TIA): No

Bleed resulted in right hemiparesis, less control, and functional outcomes in the left-limb with power: 3-/5.

He was discharged from hospital ward after 14 days.

Recommendation at discharge: Medicines and Physiotherapy

Details of Medication from onset to completion of VR therapy: anti-hypertension (Telma H, Amlog), anti-diabatic (*Glyciphage, Amaryl*), anti-cholesterol (*Atorvas*), anti-epileptic (*Gabapin*)

Physiotherapy: Deep breathing exercise, Postural drainage, Huffing or coughing, Limb positioning, Passive stretching for U/L and L/L, Passive range of motion exercises for U/L and L/L, Ankle-toe movements, Active turning in bed (supine to sit- sitting- sit to stand)

S1.2. Between 2020-2021:

In addition to the physiotherapy recommended, the patient started ayurvedic treatment after 1.5 months of his discharge from hospital after the stroke onset, and continued it for 6 months. The treatment included massage of ayurvedic oil (named as "*Prasarni Taila*", manufactured by Baidyanath and available in market) over the affected limb twice a day. Thereafter, he did not undergo any ayurvedic treatment, at the time of enrolment in this study until the completion of the intervention protocol he was not taking any ayurvedic medication.

Home Based Exercise Program- Active range of motion for U/L & L/L followed by adding free weights in graded manner, Active stretching of U/L & L/L, Static quadricep and hamstring exercises, walking for 10-15 minutes, Heel to toe walk, Heel and toe standing, sit to stand from a chair, Weight shifting in sitting and standing, bridging exercise.

S1.3. Details at the time of Enrollment (November 2021) (Other than in Manuscript):

Chronicity: 13 months

Neck Control – Normal *Trunk Control* - Normal

Manual Muscle Test (MMT for EDC muscle): 3-

Hypertonia at EDC muscle: 0

Gait: Hip hiking, foot drag *DTR* 3+ for Brachioradialis

Visual Analog Scale (VAS) for pain: 5 *Mini Mental Examination Score:* 30

Cerebellum:

Finger to Nose test (FNT): Tremor present *Heel to Shin test (HTS):* Tremor absent

Sensation:

Superficial (pain / touch / temperature / pressure) - Present

Deep (proprioception / kinesthesis / Vibration) - Present

Cortical (Tactile Localization / Stereognosis / Graphesthenia / Barognosis / 2-point discrimination / Texture recognition) - Present

Upper Limb Fugl Meyer Score:

Upper extremity: 31/36, Wrist: 8/10, Hand 14/14, Coordination Speed: 4/6, Sensation: 12/12, Passive Joint Motion: 20/24, Joint Pain: 17/24

Lower-Limb Fugl Meyer Score:

Lower Extremity: 24/28, Coordination/ speed: 6/6, Sensation: 11/12, Passive Joint Motion 19/20, Joint Pain: 20/20

Stroke Impact Scale (SIS):

Strength: 40/100, Memory and thinking: 77.1/100, Emotions: 68.8/100, Communication: 80/100, ADL/IADL: 56/100, Mobility: 67.6/100, Hand function: 64/100, Social Participation: 40/100, Stroke Recovery: 70/100

Addenbrooke's Cognitive Examination – III (ACE-III) Hindi Version:

Attention: 17/18, Memory: 23/26, Fluency:14/14, Language: 26/26, Visuospatial: 16/16

Multi-dimensional Health Locus Control (MHLC):

Form A: Internal (35/36), Chance (9/36), Powerful Others (36/36)

Form B: Internal (31/36), Chance (17/36), Powerful Others (15/36)

Form C: Internal (28/36), Chance (15/36), Doctors (16/18), Other people (11/18)

S1.4. Details of physiotherapy suggested at the time of enrollment:

- Tasks specific to upper extremity included:
    - Active assisted shoulder flexion exercise- 10 sec hold × 8 reps
    - Active shoulder abduction without elbow bending- 10 sec hold × 8 reps
    - Shoulder shrug, shoulder protraction and retraction- 10 sec hold × 8 reps
    - Active forearm pronation & supination over the table with end range stretch during supination.

- ➢ Active stretching of long wrist flexors with the hold of 30 sec followed by relaxation
- ➢ Wrist curls & extension with forearm supported at 90 degrees angle- 10 reps (3 sets)
- ➢ Fist making in prone and thrust release-30 reps (3sets)
- ➢ Fist making in mid prone position -30 reps (3sets)
- ➢ Ball squeezing and release -30 reps
- ➢ Finger opposition and release-30 reps
- ➢ Lock and key movements -30 reps
- ➢ Muscle facilitation techniques like stroking, brushing, icing - 3-5 strokes twice in a day
- ➢ Task-oriented training (TOT); Action of reaching & grasping a bottle, holding different shape objects, lifting a glass of water and flipping pages of album, pen holding & spinning, brushing, practice fastening of a zip, bottle opening, opening door/Turning a doorknob with affected hand.

- The task was made challenging by adding gradations like:
  - ➢ Gross to fine movements
  - ➢ Static to dynamic positioning
  - ➢ Single to dual activity
  - ➢ Non-weighted to the incorporation of weights
  - ➢ Indoor to the outdoor environment

- If the patient experiences any kind of pain/fatigue in wrist/hand, proximal joints like shoulder/elbow; balance or coordination difficulties while doing a particular task, the task was fragmented in small segments for the ease of practice and was gradually built up from there for achieving the required functional goal.

S1.5. Details at the time of post-therapy (other than in Manuscript):

<u>Upper Limb Fugl Meyer Score:</u>

Upper extremity: 31/36, Wrist: 10/10, Hand 14/14, Coordination Speed: 6/6, Sensation: 12/12, Passive Joint Motion: 21/24, Joint Pain: 20/24

<u>Lower-Limb Fugl Meyer Score:</u>

Lower Extremity: 25/28, Coordination/ speed: 5/6, Sensation: 12/12, Passive Joint Motion 20/20, Joint Pain: 20/20

<u>Stroke Impact Scale (SIS):</u>

Strength: 70/100, Memory and thinking: 71.4/100, Emotions: 75.5/100, Communication: 77/100, ADL/IADL: 64/100, Mobility: 75.5/100, Hand function: 76/100, Social Participation: 28/100, Stroke Recovery: 80/100

<u>Addenbrooke's Cognitive Examination – III (ACE-III) Hindi Version:</u>

Attention: 18/18, Memory: 25/26, Fluency:14/14, Language: 26/26, Visuospatial: 16/16

<u>Multi-dimensional Health Locus Control (MHLC)</u>:

Form A: Internal (25/36), Chance (17/36), Powerful Others (30/36)

Form B: Internal (29/36), Chance (12/36), Powerful Others (30/36)

Form C: Internal (26/36), Chance (19/36), Doctors (17/18), Other people (14/18)

S2. VR tasks

S2.1. Virtual Environment:

In the virtual environments developed, a stimulus (3D duck model) is presented as a target to reach within a specified time limit. Different predefined 3D models in the form of obstacles are present on the driving path. In each task, the participant was required to reach the pre-defined target position within a given time limit, avoiding collision with the obstacles. The movements performed through the joystick are projected into the 3D environments, which causes a virtual motion of the 3D car object present in the 3D world. Colliding with obstacles creates an error, and the participant gets an error notification through audio feedback. Yaw movements of the joystick drive the car forward or backward, corresponding to wrist radial/ulnar deviation, and pitch angle turns the car left or right corresponding to wrist flexion/extension. Before starting the VR task, the patient was given one practice session of 5–10 min to make themselves comfortable with handling the joystick and to familiarize themselves with virtual environment setup. All the relevant instructions regarding the VR task were made visible to the participants before starting each task. At the end of each level, the performance level is displayed quantitatively (score) and qualitatively (progress bar). Appropriate visuals as per task performance, such as "You have done well! Congratulations" or "Try better next time! Please do well", are displayed to motivate the user at this point.

S2.2. Task Description:

Two types of task environments were created i.e., Individual Environment (IE) and Combined Environment (CE). IE tasks were developed over different 3D tracks to introduce various difficult elements of the task i.e., time-limit, number of obstacles and shape of tracks. CE tasks were developed over the exact shapes of the 3D tracks used in IE, but incorporated with multiple difficulty elements simultaneously to make the tasks more challenging and permit the subjects to use the experience gained from IE tasks. This has been explained in Table S1. In the rehabilitation aspect, these tasks can be categorized into motor and motor + cognitive tasks. Motor tasks require the wrist and finger movements only. Tasks of IE modules 1–5 (Supplement Table S1) focus on motor tasks. Motor + cognitive tasks require attention, memory and planning execution ability in addition to wrist and fingers motions. Tasks of IE module-6 require the subject to remember a particular sequence of buttons (instructed before task initialization) and to press the joystick buttons in the exact sequence to remove the obstacles. Pressing incorrect button records an error. Three different maze tracks were designed in IE module 7, for which the subject has to decide the correct path by viewing the top view of the 3D world to reach the target.

Supplement Table S1: Description of VR task difficult levels

IE task levels			
**Modules**	**Levels**	**Time limit specified (s)**	**Description**
IE 1	L1 (Track 1a)<br>L2 (Track 1b)	70<br>70	Number of obstacles increased. Track width of 1a = 7 units, 1b = 9 units
IE 2	L1-L4 (Track 1b)	50, 45, 40, 35	Keeping obstacles same, time limit decreased with levels
IE 3<br>IE 4<br>IE 5	L1-L2 (Track 2)<br>L1-L2 (Track 3)<br>L1-L2 (Track 4)	45<br>60<br>90	In L2, button pressing action was added keeping obstacles same as in L1.
IE 6	L1-L5	70	Remember a random number sequence and press the buttons in that sequence

IE 7	L1-L3	70	Three different maze tracks. User has to find the correct path to reach target
**CE task levels**			
CE 1	L1-L3 (Track 1a)<br>L1-L3 (Track 1b)	65, 60, 55<br>65, 60, 55	Increase in number of obstacles and decrease in time limit
CE 2<br>CE 3<br>CE 4	L1-L4 (Track 2)<br>L1-L4 (Track 3)<br>L1-L4 (Track 4)	55, 50, 55, 50<br>70, 65, 70, 65<br>90, 85, 90, 85	Different combinations of buttons to remember and press accordingly with increased number of obstacles and reduced time limit
CE 5	L1-L2 for each maze track	70	Different combinations of buttons to remember and press accordingly with increased number of obstacles

Note: IE: Individual Environment, CE: Combined Environment, L1: Level 1 and so on.

S3. Data Acquisition:

S3.1. Details of data acquisition through Transcranial Magnetic Stimulation (TMS):

P was compliable with Transcranial Magnetic Stimulation (TMS).

- **Patient Position:** The patient sat comfortably in a relaxed condition on the chair, with forearm pronated, keeping elbow-joint at 90–120° flexion, wrist-joint at a neutral position, and fingers at rest.
- **Electrodes:** The disposable gel-based wet Ag/AgCl surface electrodes were used (bipolar configuration). The active surface-electrodes were placed on the Extensor Digitorum Communis (EDC) muscle with inter-electrode distance of 20 mm (center-to-center). Ground electrode was placed on the lateral epicondyle. Muscle contraction causing extension of third digit of hand was observed for identification of muscle-belly and electrode placement. Electrodes were connected to the EMG amplifier connected with TMS (Magstim Rapid^2, Magstim, UK).
- **Hotspot localization:** Specific brain hotspot area for the EDC (Extensor Digitorum Communis) muscle was localized. Single-pulse TMS stimuli at 100% motor threshold (MT) were applied with the procedure widely used [1]. A flat 70 mm figure-of-eight coil (type-D70 (AC), serial no. 0326, Magstim Rapid^2, UK) was used to deliver TMS stimuli. The coil was placed tangentially with handle pointing towards back, approximately 90° to the line of central sulcus and 45° away from mid-line for trans-synaptical activation of the cortico-spinal tract [1]. TMS stimuli were delivered by moving the coil in millimeters in all directions until the hotspot, producing maximum MEP (Motor Evoked Potential) response, was localized.
- **RMT** (for target muscle at rest) is defined as the minimum intensity of TMS at the hotspot required to evoke a reliable EMG response (>50 µV peak-to-peak) in at least 5 out of 10 consecutive trials [1]. After initial exploration, with 2% increments in the stimulator output, starting below threshold, RMT was determined from the optimal site and is reported as a percentage of maximum stimulator output (MSO). Then, the stimulator output is lowered in steps of 1% until there are 5 consecutive responses out of 10 trials. Each pulse was given at an interval between each stimuli of > 5 sec [1]. Five MEP signals out of ten consecutive trials were averaged.

S3.2. Magnetic Resonance Imaging Acquisition and Pre-processing:

fMRI and T1-MRI Acquisition: Structural T1 images (170 slices) and functional MRI (fMRI) BOLD images (45 sagittal ascending slices) were acquired for affected and unaffected hand movement, using 3T MR-scanner (Philips Ingenia 5.7.1, M/s. Philips Healthcare). BOLD fMRI with repetition time (TR): 1000ms, field of view (FOV): 230×230×135 mm,

flip angle 90 degrees, voxel size: 3×3×3 mm, and echo time (TE): 25ms were acquired. A MR-compatible 20″ LCD monitor (Esys in vivo eprime 1.1) was used for projection on mirror attached to head coil, where image-of-hand represents the active-block and cross on image-of-hand represents the rest-block (each block consists of 30sec with 5 times repetition). Patient performed self-initiated sequential-maximum extension and flexion task using wrist in accordance with the block-design paradigm using the affected and unaffected hand (separately).

Pre-processing of fMRI data: The pre-processing steps include realignment by aligning images to mean-image, co-registration using T1-image, normalized and smoothing with Gaussian kernel (8 × 8 × 8 Full Width at Half-Maximum) filter on pre- and post-therapy BOLD acquisitions. Talairach-client was used to correlate MNI-coordinates with gray and white matter. The BOLD signal was convolved with a canonical hemodynamic response function, and a general linear model was used to contrast all the volumes acquired during movement blocks with those acquired during rest blocks (Movement - Rest). Voxel level threshold was set to $p < 0.05$ (FWE-corrected) and cluster size threshold was set to be 5 voxels. The number of activated voxels and LI values were calculated from the masked regions of pre- and post-central gyrus, cerebellum, and SMA for affected and unaffected hemisphere separately.

DTI data acquisition: DTI data were acquired pre- and post-therapy using the same scanner with TR/TE: 4820/92 ms, Flip-angle: 90 degrees, FOV: 230×230×148 mm, voxel size: 2×2×2 mm, slice thickness: 2mm, 32 volumes with b=800 s/mm^2 and one volume with b=0 s/mm^2.

DTI data processing: DTI data was analyzed using FSL tool[1]. The diffusion toolbox "FDT" was used to pre-process the DTI images[2]. First, we corrected DTI images for "eddy current" and then applied a "dtifit" algorithm using FMRIB's Diffusion Toolbox[3] to obtain a diffusion tensor model value at each voxel. Anatomical labels were determined with FSL Atlas Query[4]. Then binary masks of specified structures containing clusters of significant voxels were calculated using Havard-Oxford sub-cortical structural atlas[5]. Further, the mean FA values were extracted from a group of voxels that may identify regions with significantly different fiber microstructure. FA is a unit-less measure indicating the directionality of water diffusion which is 0 in a liquid compartment (fully isotropic) and becomes near to 1 in dense tracts (unidirectional diffusion). Interhemispheric FA asymmetry (aFA) values ranging from −1 to +1 were calculated for each of the regions using the formula [2]:

$$\text{FA asymmetry (aFA)} = \frac{(FA_{contralesional} - FA_{ipsilesional})}{(FA_{contralesional} + FA_{ipsilesional})}$$

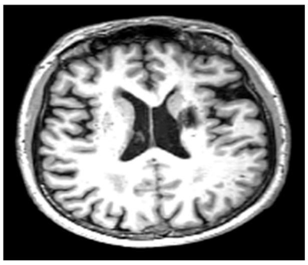

Supplementary Figure S1: Anatomical (T1) MR image of patient at enrollment

---

[1] "FSL - FslWiki." https://fsl.fmrib.ox.ac.uk/fsl/fslwiki (accessed Apr. 03, 2022).

[2] "FDT/UserGuide - FslWiki." https://fsl.fmrib.ox.ac.uk/fsl/fslwiki/FDT/UserGuide (accessed Apr. 03, 2022).

[3] "FDT - FslWiki." https://fsl.fmrib.ox.ac.uk/fsl/fslwiki/FDT (accessed Dec. 21, 2022).

[4] "Atlasqueryy - FslWiki." https://fsl.fmrib.ox.ac.uk/fsl/fslwiki/Atlasqueryy (accessed Apr. 03, 2022).

[5] "Atlases - FslWiki." https://fsl.fmrib.ox.ac.uk/fsl/fslwiki/Atlases (accessed Dec. 21, 2022).

3D tractography of Cortico-spinal tract (CST) using DTI: The 3D tractography were generated using the console of Phillips Ingenia 5.7.1. It was observed that (Main text Figure 4), post-therapy CST of ipsilesional (right) hemisphere have been denser and more intact tract as compared to the pre-therapy (indicated by red arrow mark). This is in support of the fact that FA-asymmetry (aFA) value for CST has been reduced from Pre-therapy values (0.005) to post-therapy values (−0.012), indicating better integrity of the tracts.

S3.3 Task-specific Performance Measures

In each task level, time taken to complete the task, 3D-coordinates of path travelled and the errors occurred were recorded for further analysis. Subjective trajectory plot along with three task-specific performance metrics were evaluated from the recorded data and explained below:

i. **Time taken to complete the task (TCT):** Time taken to complete the task is defined by:

$$\text{TCT} = \frac{\textit{Time taken to complete the task}}{\textit{Specified time limit for the task}} \times 100\ (\%)$$

The parameter TCT was used to examine whether the subject could complete a specific task within a reasonable time interval. An appropriate and reasonable time limit was set for each particular task level in order to complete the task. Values of TCT less than 100% indicate that the subject was able to complete a particular task successfully within a given time limit. Values of TCT more than or equal to 100% indicates that the subject was slow to achieve the target within the specified time. A decrease in TCT over the sessions is a success indicator that will indicate better learning and execution of tasks.

ii. **Relative percentage error:** Relative percentage error is defined by:

$$\text{Relative percentage error} = \frac{\textit{Ideal distance of track} - \textit{Distance covered by the subject}}{\textit{Ideal distance of track}} \times 100(\%)$$

The relative % error parameter gives a numerical representation of the distance covered by the subjects to reach the target from the initial position. High values of % relative error indicate that the subject has traveled more distance than the ideal distance of the track designed, and vice-versa. This might be due to the tendency of the subject to deviate from the ideal path because of poor precision to control the car or off shooting the track. A reduction in relative percentage error during the session will indicate better motor control and coordination.

iii. **Smoothness of trajectory:** Smoothness of trajectory is defined by:

$$\text{Smoothness of trajectory} = \sqrt{\frac{1}{2}\int\left(\left(\frac{d2x}{dt2}\right)\right)^2 + \left(\frac{d2y}{dt2}\right)\right)^2 + \left(\frac{d2z}{dt2}\right)\right)^2\right)\ \frac{t^5}{s^2}}$$

where ‘x’, ‘y’, ‘z’ represents the 3D coordinates of trajectory path, ‘t’ is the motion time, and, ‘s’ is the motion distance. The lower the value of this parameter, the smoother is the motion trajectory, and the shorter will be the motion time, indicating improvement in the subject’s ability to control and coordinate the 3D motion.

iv. **Trajectory Plot:** These are the plots of coordinates of motion path obtained at an interval of one second. Trajectory plots represent the variation in the shape of the path traveled for a particular duration of time. It can indicate the task performance by providing the path traveled by the subject for a particular task level and can be used to analyze whether the subject was able to complete the track or if any wrong trajectory was followed [3].

The x- and y-axis of the trajectory plots represent the recorded X and Z—coordinates of the traveled path respectively. The Y—coordinate values remain fixed throughout the trajectory (In the default coordinate system of the Vizard software environment, the Y coordinate value represents height.).

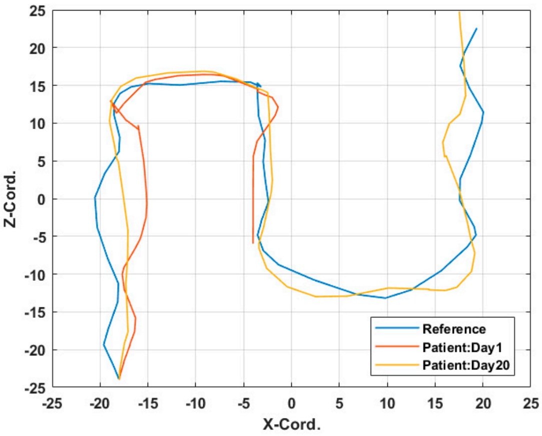

Supplementary Figure S2 representing the patient's trajectory at day 1 and day 20 for another task level CE3-L1 in comparison to a representative healthy subject's trajectory.

Supplement Table S2. Comparison of performance metrics obtained from the patient (P) at day 1, 10 and 20 with the mean of forty healthy subjects (Reference)

Performance Metrics	Patient (P)			Mean of forty healthy subjects (Reference)
	Day 1	Day 10	Day 20	
**TCT (%)**	100	88.6	68.4	71.2
**Smoothness of trajectory**	2783.2	1862.7	648	659
**Relative percentage error (%)**	33.8	9.9	6.5	6.9

S3.4 Subjective Questionnaire Feedback (SQF):

A self-designed subjective questionnaire form (SQF) has been administered to record the patient's experience regarding the usability of the joystick and VR tasks. The SQF focused on the patient's interestingness, and controllability of the VR tasks, user-friendliness, pain/fatigue/adverse event experienced, individual suggestions for further improvement, etc.

Supplement Table S3. Subjective questionnaire feedback (SQF)

No.	Questions	Patient (P)
1	Were the tasks simple and interactive?	Yes

2	Were you able to understand the procedure and synchronize with it clearly?	Yes
3	Did you experience any tiredness or fatigue during the protocol duration?	No (experienced at initial period of the therapy)
4	Do you find the joystick easy and comfortable to operate after trial session?	Yes
5	Did you face any difficulties while understanding the task instructions?	No
6	Did you find the tasks interesting, engaging and motivating?	Yes
7	Was the task GUI interesting?	Yes (Top view pane size to be increased for a better visibility)
8	Is the goal of task clear, engaging and interesting?	Yes
9	Do you want to perform the tasks in future?	Yes
10	Are the text instructions, buttons and time counter clear and readable?	Yes
11	Does the lag between real and virtual movements acceptable?	No lag experienced
12	Any visual discomfort perceived during session?	No
13	The rest between the task levels is fine?	Yes
14	Any postural fatigue (excluding arm) felt?	No (at initial period, fatigue at shoulder was experienced)
15	Do you find the difficulty levels too difficult to execute?	No
16	Do you find the cognitive tasks too difficult to execute?	No
17	Was controlling the virtual movements with joystick easy?	Yes
18	Any adverse event experienced?	No
19	Do you feel it safe to use?	Yes
20	Did trajectory thickness and the panel size allow good playability of the game?	Yes
21	Were you able to use the wrist and fingers as promptly as required?	Yes (at initial period, pain at fingers, gradually vanished)
22	How many VR sessions or how much duration it took to be comfortable?	4 sessions
23	Was the audio-visual feedback useful and interesting?	Yes

24	Do you feel your attempt is proportional to the task executed in going straight, back, taking left and right turns?	Yes
25	Is the duration of the tasks fine – long /short?	Fine
26	Do you think you can use the setup at home?	Yes (provided all instructions on how to operate)
27	What did you like in the VR sessions?	Interesting tasks to perform as compared to exercise tasks<br>Could perform tasks for a long period at a time without boredom<br>GUI and 3d object interaction interesting
28	What did you dislike in the VR sessions?	Use of similar environments and objects<br>Joystick was a bit uncomfortable to be used with left arm when used first time
29	Did you have VR exposure earlier?	No
30	Is there anything you would like to change in the session?	Use of new 3d objects and audio-visuals<br>Increase the size of top view window<br>Buttons of joystick to be placed at more comfortable position
31	Do you prefer doing these virtual tasks over real-world exercise tasks?	Yes
32	Do you feel any improvement after involving your hand in the tasks?	Yes (Smoother motions of fingers without any pain)
33	Did you feel you could prevent the involvement of elbow and shoulder joints?	No
34	How satisfied you were with the treatment? (1-5) [1-lowest score, 5-highest score]	4
35	Did you feel the hand stiffness increased during the attempt or the execution of the tasks?	Initially experienced at fingers, later vanished

References

[1] P. M. Rossini *et al.*, "Non-invasive electrical and magnetic stimulation of the brain, spinal cord, roots and peripheral nerves: basic principles and procedures for routine clinical and research application. An updated report from an IFCN Committee," *Clin. Neurophysiol.*, vol. 126, no. 6, pp. 1071–1107, 2015.

[2] C. M. Stinear, P. A. Barber, P. R. Smale, J. P. Coxon, M. K. Fleming, and W. D. Byblow, "Functional potential in chronic stroke patients depends on corticospinal tract integrity," *Brain*, vol. 130, no. 1, pp. 170–180, 2007.

[3] A. Alamri, M. Eid, R. Iglesias, S. Shirmohammadi, and A. El Saddik, "Haptic virtual rehabilitation exercises for poststroke diagnosis," *IEEE Trans. Instrum. Meas.*, vol. 57, no. 9, pp. 1876–1884, 2008, doi: 10.1109/TIM.2008.919878.
